# Rapid deterioration of renal function following coronavirus disease 2019 in a renal transplant recipient

**DOI:** 10.1002/iju5.12566

**Published:** 2022-12-16

**Authors:** Seigo Machiya, Shigeaki Nakazawa, Shota Fukae, Ryo Tanaka, Ayumu Taniguchi, Kazuaki Yamanaka, Shiro Takahara, Tomoko Namba‐Hamano, Ryoichi Imamura, Norio Nonomura

**Affiliations:** ^1^ Department of Urology Osaka University Graduate School of Medicine Suita Osaka Japan; ^2^ Kansai Medical Hospital Renal Transplantation Clinic Toyonaka Osaka Japan; ^3^ Department of Nephrology Osaka University Graduate School of Medicine Suita Osaka Japan

**Keywords:** acute kidney injury, acute tubular necrosis, COVID‐19, graft biopsy, renal transplantation

## Abstract

**Introduction:**

The coronavirus disease 2019 pandemic emerged in December 2019. Renal transplant recipients receiving chronic immunosuppression are considered to be at a high risk of infection. Aside from upper respiratory tract symptoms, coronavirus disease 2019 has also been reported to cause acute kidney injury in 20–50% of infected cases.

**Case presentation:**

A 62‐year‐old male renal transplant recipient presented with high fever, diarrhea, and cough, concurrent with rapid deterioration of graft function. The patient tested positive for coronavirus disease 2019. The pathological findings of the graft biopsy revealed diffuse flattening of tubular epithelial cells and extensive loss of the brush border in proximal tubular cells. Mycophenolate mofetil was discontinued and sotrovimab, remdesivir, intravenous immunoglobulin, and intravenous methylprednisolone were administered, resulting in gradual improvements in clinical symptoms and renal function.

**Conclusion:**

We describe a case of a coronavirus disease 2019‐infected kidney transplant recipient who developed severe acute kidney injury caused by severe acute tubular necrosis.

**Abbreviations & Acronyms**
AKI: acute kidney injury
BT: body temperature
COVID‐19: coronavirus disease 2019
CRP: C‐reactive protein
CT: computed tomography
ICU: intensive care unit
IVIg: immunoglobulin
MMF: mycophenolate mofetil
sCr: serum creatinine
SpO_2_
: saturation of percutaneous oxygen

**Keynote message**

A renal transplant recipient manifested rapid graft function deterioration due to COVID‐19. Pathological findings of the graft biopsy revealed severe acute tubular necrosis. Remdesivir, sotrovimab, intravenous methylprednisolone, and intravenous immunoglobulin therapy ameliorated clinical symptoms and improved graft function.

## Case presentation

A 62‐year‐old male underwent ABO blood‐type‐incompatible living kidney transplantation in November 2020. Diabetic nephropathy is the primary cause of end‐stage renal disease. Maintenance immunosuppressive therapy comprised tacrolimus, MMF, everolimus, and prednisolone. He received two doses of the COVID‐19 messenger ribonucleic acid vaccine in July 2021.

**Fig. 1:**
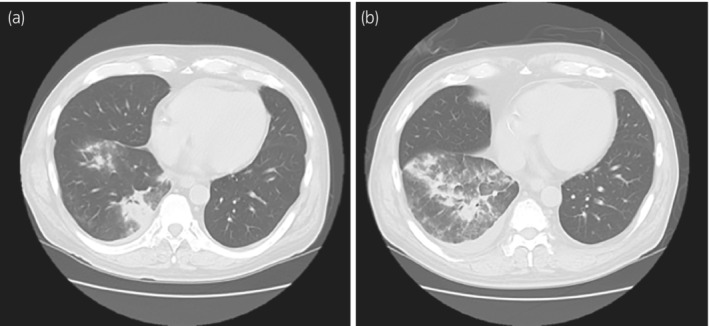
Thoracic CT (a) at disease onset and (b) 4 days after onset.

**Fig. 2:**
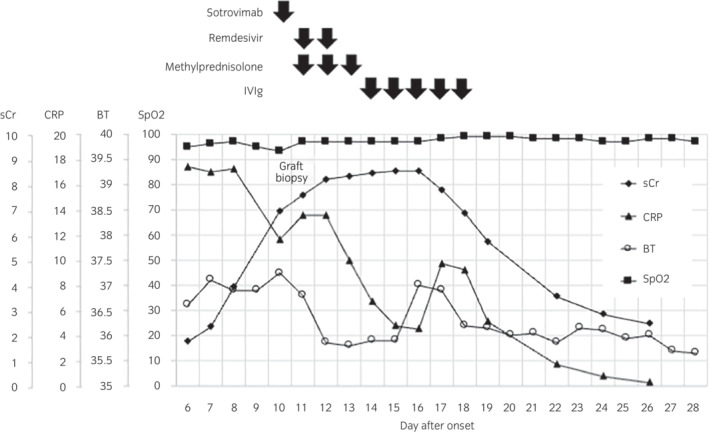
Clinical course of the patient.

In May 2022, the patient was admitted to our affiliated hospital with the chief complaint of high fever and watery diarrhea persisting for 5 days. Thoracic CT revealed an irregular frosted glass area in the right lower lung (Fig. 1a). As both polymerase chain reaction and antigen tests were negative for COVID‐19 on admission, tazobactam and piperacillin were initiated following a diagnosis of infectious enteritis and bacterial pneumonia. MMF was discontinued, and other immunosuppressive doses were maintained according to their blood concentrations. Four days of antibiotic therapy failed to resolve the symptoms, and a repeat CT showed aggravation of pneumonia (Fig. 1b). In addition, the patient's renal function deteriorated rapidly in this short period, from a sCr level of 1.42 mg/dL at baseline to 6.93 mg/dL (Fig. 2). The patient was referred to our hospital on day 10 for further examination and treatment.

On admission, the patient was re‐tested for COVID‐19, this time showing a positive result. He presented with a wet cough and high fever and complained of fatigue and shortness of breath. Although his SpO_2_ level decreased to 92.3%, oxygen administration was unnecessary. Sotrovimab (500 mg/body weight) and remdesivir (200 mg loading dose, followed by 100 mg) were administered; however, renal function continued to deteriorate rapidly (sCr 7.59 mg/dL), and doppler ultrasonography of the graft showed a significant loss of diastolic blood flow. As acute rejection was suspected, graft biopsy and single‐antigen panel‐reactive antibody bead tests were performed. As an empirical treatment, steroid pulse therapy (methylprednisolone 500 mg/body, 3 days) followed by low‐dose IVIg treatment (100 mg/kg, 5 days) was administered. The patient's diabetes was poorly controlled with an HbA1c of 8.0% and was strictly controlled with insulin.

**Fig. 3:**
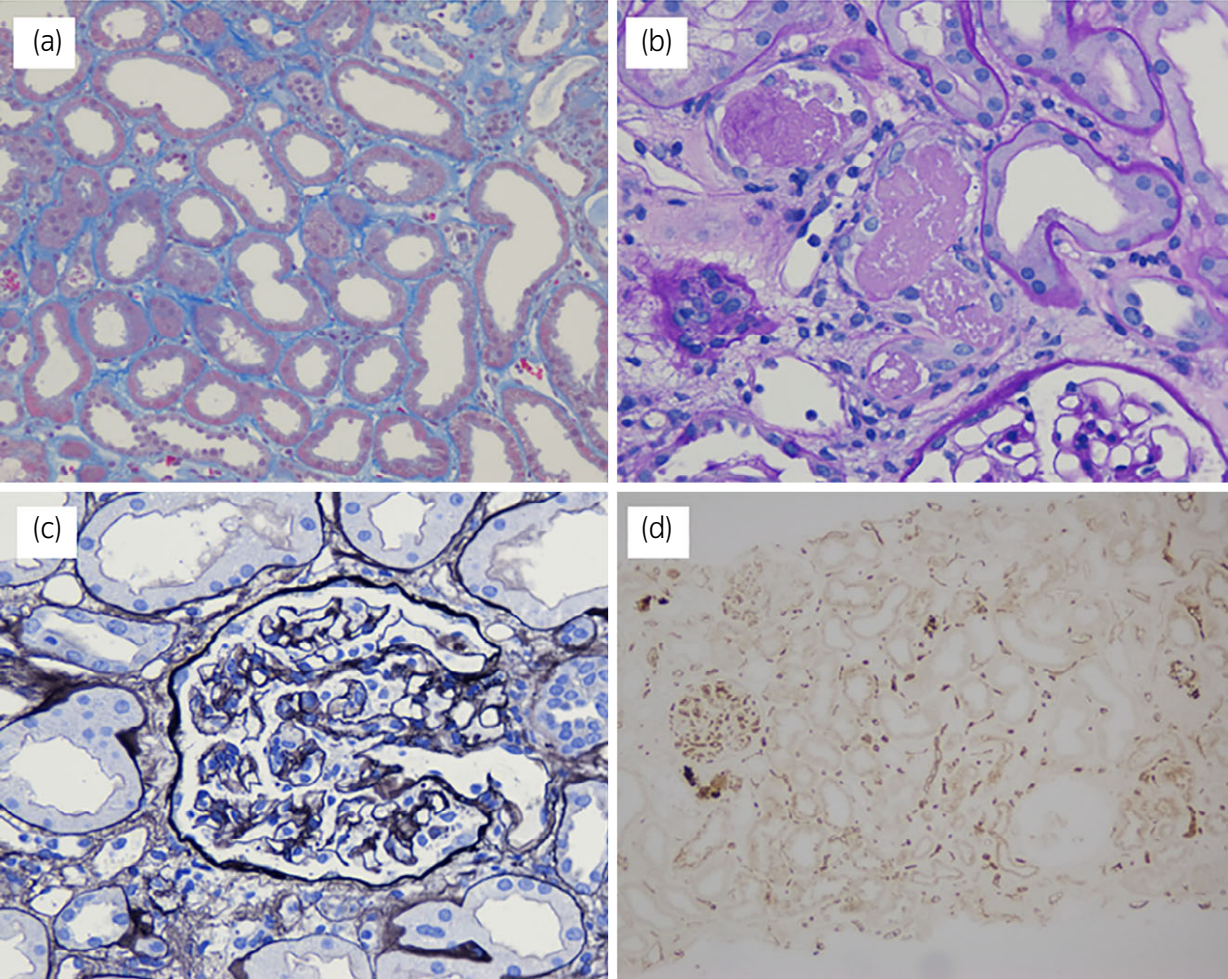
Pathological findings of graft biopsy. (a) diffuse flattening of the tubular epithelial cells, (b) cellular debris and cast formation within the lumina, (c) a glomerulus with segmental shrinkage of the tuft and wrinkling of the basement membranes, (d) diffuse positive C4d staining in the peritubular capillary without inflammation.

Pathological examination of the graft biopsy revealed diffuse flattening of the tubular epithelial cells and extensive loss of the brush border in proximal tubular cells (Fig. 3). Cellular debris and cast formation were observed within the lumina; however, thrombi were not observed in the glomeruli. Some glomeruli showed segmental shrinkage of the tuft with wrinkling of basement membranes. No findings were related to rejection. Immunohistochemical analysis revealed diffuse positive C4d staining in the peritubular capillary without inflammation, which was compatible with ABO‐incompatible transplantation. We did not observe any other specific deposition on immunofluorescence staining.

Donor‐specific antibodies were not detected serologically. Although sCr level peaked at 8.53 mg/dL on the 16th day after onset, his renal function gradually improved without requiring temporary dialysis. The patient was discharged on the 28th day after onset (Fig. 2).

## Discussion

COVID‐19, caused by the severe acute respiratory syndrome coronavirus 2, was first identified in Wuhan, China, in December 2019.^1^ Since then, the disease has spread globally at an alarming rate, causing a pandemic and posing a major threat to international public health. This infection mainly presents as fever, cough, and breathing difficulties, and only a low proportion of patients develop severe symptoms.^2^

Renal transplant patients are among the most vulnerable populations to COVID‐19, and many reports discussing their high risk have been published.^3^ Various nationwide registries and multicenter studies have reported an incidence of 8.3 to 17.7 per 1000 renal transplants.^4^

COVID‐19 has been reported to cause AKI at an incidence of 20–50% in patients infected with COVID‐19.^3^, ^5^, ^6^, ^7^, ^8^ Importantly, AKI was also significantly associated with mortality in both general hospitalized patients and patients admitted to ICUs.^9^, ^10^ The possible mechanisms of COVID‐19‐associated AKI have been pointed out from the beginning, including unstable circulation, cytokine storms, and direct viral invasion.^11^ Despite descriptions of COVID‐19 as a cytokine storm syndrome, levels of circulating cytokines are often lower in patients with COVID‐19 than in patients with acute respiratory distress syndrome with causes other than COVID‐19. A recently published report on renal biopsies of 10 COVID‐19 patients with AKI did not detect the severe acute respiratory syndrome coronavirus 2 (SARS‐CoV‐2) virus in renal tissue.^12^ Therefore, it is not possible to conclude whether the SARS‐CoV‐2 virus can directly infect tubular epithelial cells or podocytes.

From the analysis of kidney biopsy samples from 17 patients with AKI, acute tubular injury, collapsing glomerulopathy, and endothelial injury or thrombotic microangiopathy were the most common histological findings.^13^ Regional inflammation and endothelial injury also have been reported.^14^ Based on single‐cell transcriptome analysis, cytopathic effects of SARS‐CoV‐2 on podocytes and proximal straight tubule cells may cause AKI in patients with COVID‐19.^15^ In the present case, there was no evidence of acute rejection or micro thrombosis, although severe acute tubular necrosis was observed. We thus concluded that the cause of deteriorated renal function was AKI associated with COVID‐19.

Prior reports have indicated that kidney transplant recipients with COVID‐19 have higher tacrolimus levels in their blood than non‐infected kidney transplant recipients.^16^ Although the tacrolimus level is also often elevated in diarrhea, it was markedly decreased in this case (trough level 1.8 mg/dL). This event required the consideration of both AKI due to COVID‐19 and acute rejection as possible causes of deteriorated renal function until a pathological diagnosis could be made.

Sotrovimab and remdesivir have been reported to significantly reduce mortality due to COVID‐19, and have no apparent nephrotoxic effects on renal transplant patients.^17^, ^18^ However, sufficient evidence has not yet been accumulated regarding steroid pulse therapy for COVID‐19 in renal transplant recipients. In a retrospective cohort study, treatment with methylprednisolone was shown to reduce the risk of death in patients with acute respiratory distress syndrome.^19^ In this case, steroid pulse therapy and IVIg did not appear to have an adverse effect on the treatment of COVID‐19. As no specific treatment options exist for AKI secondary to COVID‐19, multidisciplinary treatment for COVID‐19 may result in the improvement of AKI.

Grade 3 AKI is reported to occur in 30% of patients requiring ICU admission but in only 10% of patients not requiring ICU admission.^20^ Our patient had moderate disease without respiratory failure according to the COVID‐19 severity classification, but his renal function deteriorated rapidly. In conclusion, a definitive diagnosis based on renal biopsy and appropriate treatment resulted in salvage and improvement of renal function.

## Author contributions

**Seigo Machiya:** Writing – original draft. **Shota Fukae:** Writing – review and editing. **Ryo Tanaka:** Writing – review and editing. **Ayumu Taniguchi:** Writing – review and editing. **Kazuaki Yamanaka:** Writing – review and editing. **Shiro Takahara:** Writing – review and editing. **Tomoko Namba‐Hamano:** Writing – original draft. **Ryoichi Imamura:** Writing – review and editing. **Norio Nonomura:** Writing – review and editing.

## Conflict of interest

The authors declare no conflict of interest.

## Approval of the research protocol by an Institutional Reviewer Board

Not applicable.

## Informed consent

Written informed consent was obtained from the patient for the publication of this case report and the accompanying images.

## Registry and the Registration No. of the study/trial

Not applicable.
